# Layer-Specific Shear Wave Elastography Reveals the Tissue Origin of MyotonPRO-Derived Muscle Stiffness

**DOI:** 10.3390/diagnostics16152346

**Published:** 2026-07-27

**Authors:** Sebastian Szajkowski, Michał Dwornik, Krzysztof Suszyński, Jarosław Pasek

**Affiliations:** 1 Faculty of Medical Sciences, Warsaw Medical Academy of Applied Sciences, 8 Rydygiera St., 01-793 Warszawa, Poland; sebastianszajkowski@wp.pl; 2 Medical Rehabilitation and Osteopathy Clinic REHApunkt, Traugutta 12 St., 05-820 Piastów, Poland; mtdwornik@gmail.com; 3 Department of Sports Medicine and Physiology of Physical Exercise, Faculty of Health Science in Katowice, Medical University of Silesia, 40-055 Katowice, Poland; ksuszynski@sum.edu.pl; 4 Collegium Medicum im dr Władysława Biegańskiego, Jan Długosz University in Częstochowa, 13/15 Armii Krajowej St., 42-200 Częstochowa, Poland

**Keywords:** shear wave elastography, MyotonPRO, muscle stiffness, elasticity imaging techniques

## Abstract

**Background:** Shear wave elastography (SWE) and myotonometry are commonly used to assess muscle stiffness, but they reflect different aspects of tissue mechanics. SWE allows layer-specific evaluation, whereas myotonometry captures the response of a broader tissue complex. The contribution of individual layers to MyotonPRO-derived stiffness remains unclear. This study examined the relationship between MyotonPRO stiffness and layer-specific SWE measurements in the tibialis anterior muscle. **Methods:** In this cross-sectional study, 56 healthy adults were evaluated. Muscle stiffness was assessed using MyotonPRO and SWE across three regions of interest: superficial tissues including skin, subcutaneous tissue, and deep fascia (ROI 1); muscle tissue excluding fascia (ROI 2); and a composite region including all tissues from the skin surface to a depth of 2 cm (ROI 3). Relationships were analyzed using Spearman correlation, multiple linear regression, and Bland–Altman analysis. **Results:** Moderate but consistent correlations were observed between MyotonPRO stiffness and SWE values in ROI 2 (ρ = 0.516, *p* < 0.001) and ROI 3 (ρ = 0.550, *p* < 0.001), whereas no significant association was found in ROI 1. SWE values in ROI 2 and ROI 3 were significant predictors of MyotonPRO stiffness, explaining 38% of the variance. Bland–Altman analysis demonstrated moderate agreement with a systematic bias toward higher MyotonPRO values. **Conclusions:** MyotonPRO-derived stiffness reflects a composite mechanical response of muscle and surrounding tissues, with the strongest associations observed in regions encompassing both muscle and fascia. These findings highlight the importance of tissue composition and ROI selection in stiffness assessment and may facilitate more accurate interpretation of MyotonPRO measurements in musculoskeletal diagnostics and rehabilitation.

## 1. Introduction

Shear wave elastography (SWE) is a modern imaging technique that enables quantitative assessment of the biomechanical properties of soft tissues by analyzing the propagation of shear waves generated by an acoustic impulse within a defined region of interest (ROI). This method allows for the estimation of Young’s modulus, an indirect measure of tissue stiffness expressed in kilopascals (kPa). Owing to its ability to simultaneously assess both structural and mechanical properties, SWE is considered one of the most promising diagnostic tools in musculoskeletal research [1]. Despite its advantages, the application of SWE in muscle tissue is associated with important methodological limitations. Muscle anisotropy, heterogeneous structure, and the presence of multiple tissue components (muscle fibers, fascia, connective tissue, and vessels) may lead to substantial variability in measurements within the same muscle. Additionally, factors such as measurement depth and the location of the ROI relative to adjacent structures, including bone and superficial tissues, may influence the results [2].

Portable devices for assessing mechanical tissue properties, such as the MyotonPRO (MyotonPRO, Myoton AS, Tallinn, Estonia), represent an alternative to elastography. This device uses a digital palpation method based on a brief mechanical impulse that induces damped natural oscillations of the tissue. Analysis of these oscillations enables the estimation of biomechanical parameters, including dynamic stiffness expressed in N/m. This method has been shown to be reliable and reproducible in the assessment of superficial muscles and tendons [3]. In contrast to SWE, which evaluates mechanical properties within a precisely defined ROI, MyotonPRO captures the mechanical response of the entire tissue complex within the penetration range of the mechanical impulse. Consequently, the measured stiffness value may reflect a composite response of multiple tissue layers rather than the properties of a single structure. Importantly, the two methods are based on different physical principles and provide results in different units (kPa vs. N/m), which complicates their direct comparison [4].

Previous studies have demonstrated that both SWE and myotonometric devices can be used for the quantitative assessment of skeletal muscle stiffness; however, the relationship between measurements obtained using these methods, particularly in terms of the contribution of individual tissue layers, remains insufficiently understood [4,5]. To the best of our knowledge, no previous studies have directly compared MyotonPRO measurements with SWE assessments across different ROI layers to identify the structural origin of the measured stiffness values. In particular, there is a lack of studies investigating which tissue components predominantly determine the stiffness values recorded by MyotonPRO. The tibialis anterior muscle was selected as the experimental model due to its superficial location and relatively uniform fiber architecture, which facilitate reliable and reproducible measurements using both SWE and mechanical methods. It has been shown that superficial muscles exhibit lower measurement variability, and simpler architecture reduces the impact of anisotropy on the results. Furthermore, this muscle is frequently investigated in biomechanical studies, allowing for comparison with existing literature [6].

Understanding which tissue components contribute to MyotonPRO measurements is clinically important, as the device is increasingly used in musculoskeletal rehabilitation, sports medicine, and neurology to assess muscle stiffness and monitor treatment outcomes. Clarifying whether the recorded stiffness reflects muscle tissue alone or also includes fascia and superficial tissues may improve the interpretation of measurements and support appropriate selection of assessment methods. This may facilitate more accurate evaluation of tissue-specific changes in conditions such as spasticity and musculoskeletal disorders, as well as monitoring patients’ responses to rehabilitation and other therapeutic interventions.

### Aim of the Study

The aim of this study was to determine the contribution of individual tissue structures to stiffness values measured by MyotonPRO by comparing them with SWE measurements obtained from layer-specific regions of interest within the tibialis anterior muscle. It was hypothesized that MyotonPRO-derived stiffness reflects a composite mechanical response of multiple tissue layers, and would therefore be most strongly associated with SWE measurements representing integrated tissue regions rather than isolated superficial structures.

## 2. Materials and Methods

### 2.1. Study Design and Ethical Considerations

This cross-sectional study was conducted at the Medical Rehabilitation and Osteopathy Clinic “REHApunkt” in Piastów, Poland. The study design, conduct, and reporting were carried out in accordance with the STROBE (Strengthening the Reporting of Observational Studies in Epidemiology) guidelines. The study was approved by the Bioethics Committee of the Warsaw Medical Academy of Applied Sciences, Warsaw, Poland (approval No. 202506WAM04; approval date: 30 June 2025), and was conducted in accordance with the principles of the Declaration of Helsinki. All participants were informed about the purpose and procedures of the study and provided written informed consent prior to participation.

### 2.2. Participants

Healthy volunteers were recruited for the study. Inclusion criteria included the absence of musculoskeletal complaints and a body mass index (BMI) between 18 and 25 kg/m^2^. Exclusion criteria comprised a history of orthopedic or neurological disorders, previous lower limb surgery, or the presence of pain in the assessed body regions at the time of examination. All measurements were performed under controlled laboratory conditions. Upon arrival, participants rested for 10 min at room temperature to allow for physiological stabilization, after which anthropometric parameters, including height, body mass, and BMI, were assessed.

### 2.3. Standardization of Measurements

**Figure 1:**
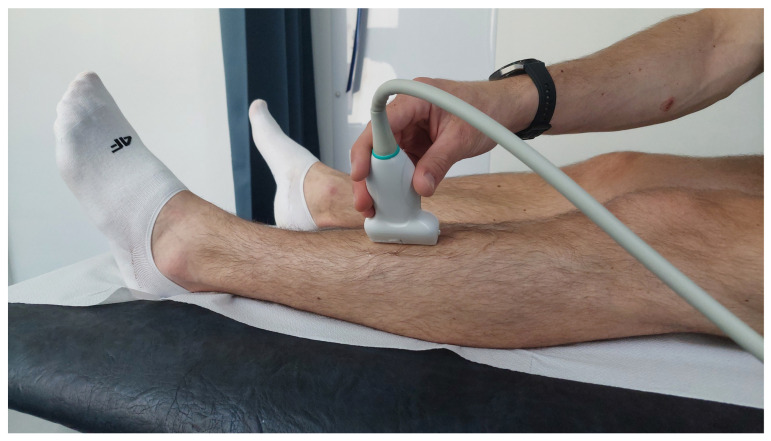
Standardized participant positioning and ultrasound transducer placement during SWE assessment of the tibialis anterior muscle.

The tibialis anterior muscle was assessed under resting conditions. All measurements were performed with participants in the supine position, with the examined lower limb fully relaxed and resting on the examination table. The ankle was positioned in a standardized resting position close to the anatomical neutral position. No additional external supports, wedges, or fixation devices were applied to the foot or ankle in order to avoid external mechanical constraints that could influence passive muscle tension. Participants were instructed to remain completely relaxed throughout the examination, and no voluntary contraction of the lower limb muscles was required during data acquisition. The standardized participant positioning and measurement setup are illustrated in Figure 1. This positioning was intended to minimize passive stretching or shortening of the tibialis anterior muscle while ensuring consistent measurement conditions across all participants [7,8].

All SWE and MyotonPRO measurements were performed during a single examination session following the initial 10-min rest period. SWE assessment was performed first, followed immediately by MyotonPRO measurements at the same predefined measurement site without repositioning the participant, thereby ensuring identical measurement conditions for both methods. The measurement site was determined based on anatomical landmarks. The distance between the inferior border of the patella and the midpoint of the ankle joint (defined between the medial and lateral malleoli) was measured, and the midpoint of this distance was identified. The measurement point was then located approximately 2 cm lateral to this line, within the muscle belly of the tibialis anterior, corresponding to its region of greatest thickness, which contributes to high measurement reliability (high intraclass correlation coefficient, ICC). This site is commonly used for the assessment of biomechanical properties of the tibialis anterior muscle using MyotonPRO, B-mode ultrasound imaging, and SWE [9,10,11]. The location of the measurement point was additionally verified using B-mode ultrasound imaging to confirm its position within the thickest portion of the muscle belly. The measurement site was marked on the skin with a dermatological marker, and all subsequent measurements were performed with reference to this point, corresponding to the center of the ultrasound probe to ensure spatial consistency between modalities. Throughout the experiment, the roles of the researchers were standardized: one operator was responsible for ultrasound system operation, a second ensured stabilization of the ultrasound probe or the MyotonPRO device, and a third recorded the measurement data. All measurements were performed by trained and experienced ultrasonographers and physiotherapists.

### 2.4. Shear Wave Elastography

SWE was performed using a Mindray DC-70 ultrasound system (Mindray Bio-Medical Electronics Co., Shenzhen, China) equipped with a high-frequency linear transducer (L12-3E, 3–12 MHz). Imaging was conducted using a musculoskeletal (MSK) preset, enabling quantitative assessment of tissue stiffness based on Young’s modulus expressed in kilopascals (kPa). During acquisition, the transducer was positioned perpendicular to the skin surface and aligned parallel to the muscle fiber orientation to minimize anisotropy-related effects. The center of the transducer was placed directly over the predefined measurement site at the midpoint of the tibialis anterior muscle belly. Acoustic coupling was ensured using ultrasound gel, and the transducer was applied with minimal pressure to avoid tissue compression. During image acquisition, the built-in M-STB (Motion Stability) Index of the Mindray DC-70 system was used to monitor image stability. The M-STB Index is a five-star quality indicator reflecting the influence of probe motion, tissue motion, and other motion-related artifacts during SWE acquisition. Only images achieving the highest M-STB stability rating (five stars) were accepted for further analysis. In addition, elastograms were visually inspected before quantitative analysis. Acquisitions showing obvious motion artifacts, incomplete or non-homogeneous shear-wave color filling within the elastographic box, or incorrect ROI placement relative to the predefined anatomical structures were discarded and reacquired. Measurement reliability was subsequently assessed using the interquartile range to median ratio (IQR/MED), and only measurements with IQR/MED < 20% were included in the final analysis. The SWE acquisition and quality-control procedures applied in the present study were based on current methodological recommendations and are comparable to recently published Mindray-based shear wave elastography protocols [12].

**Figure 2:**
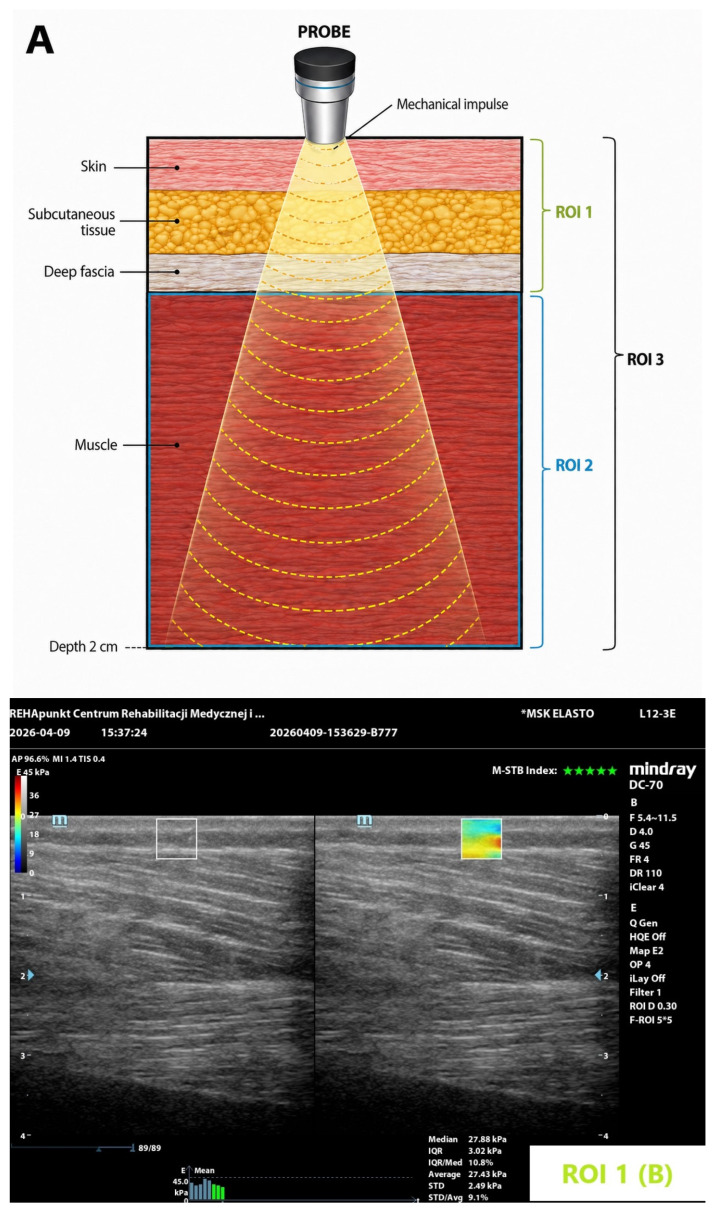

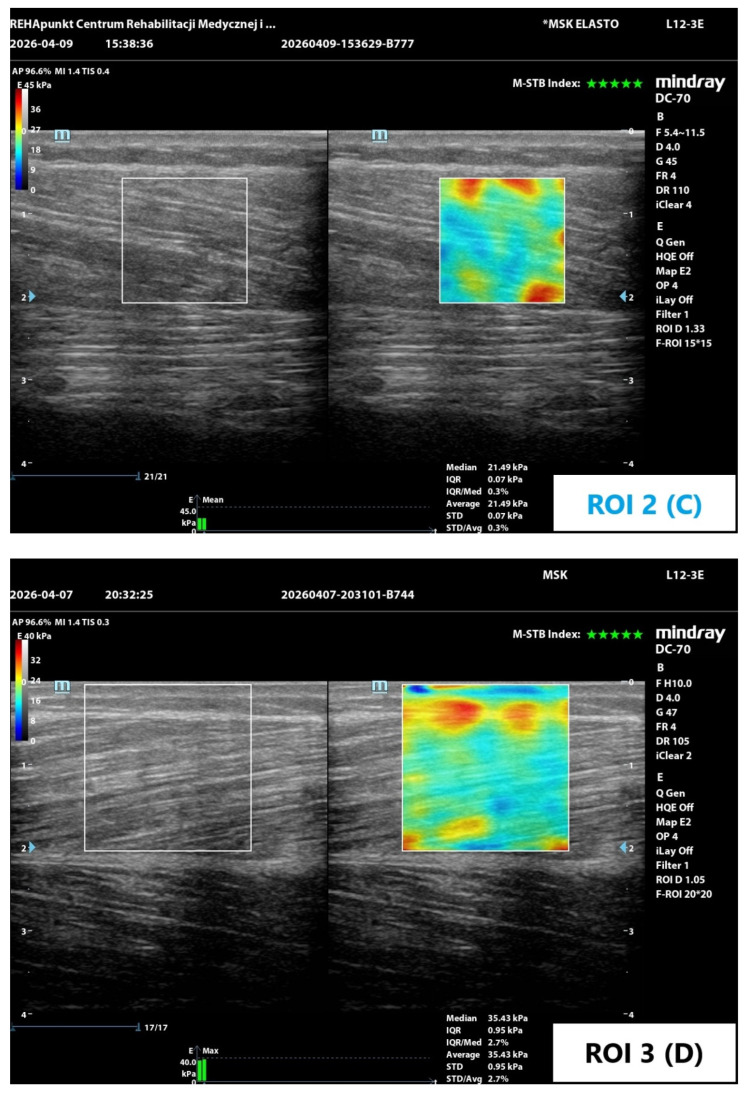
(**A**–**D**). Schematic representation (**A**) and representative ultrasound images (**B**–**D**) of tissue layers and regions of interest (ROI 1–ROI 3) used for SWE assessment of the tibialis anterior muscle. The schematic illustrates the spatial relationship between tissue layers and the depth-dependent propagation of the mechanical impulse generated by the MyotonPRO device. The defined ROIs reflect different analytical depths and tissue compositions, including a composite region corresponding to the full measurement depth (0–2 cm). Only images achieving the highest M-STB stability rating (five green stars) were accepted for analysis. (**B**–**D**) Representative ultrasound images illustrating ROI 1 (**B**), ROI 2 (**C**), and ROI 3 (**D**). (Created by the authors).

Three regions of interest (ROIs) were defined based on the range of analyzed tissue structures. ROI dimensions were adapted to individual anatomical characteristics to ensure accurate representation of the targeted tissue layers. The use of fixed ROI sizes could result in inclusion of non-target tissues, particularly in superficial regions with high inter-individual variability in thickness. In most cases, the standard ROI size was used, and adjustments were made only when required to maintain anatomical specificity. ROI 1, with a standard size of 5 × 5 mm, included superficial tissues (skin and subcutaneous tissue) as well as the deep fascia, with dimensions ranging from 3 × 3 mm to 5 × 8 mm depending on individual anatomical conditions. ROI 2 encompassed muscle tissue exclusively, extending from immediately below the deep fascia to a depth of 2 cm, thereby minimizing fascial influence. Intramuscular aponeuroses and other connective tissue structures were carefully excluded to ensure analysis of homogeneous muscle tissue. ROI placement was guided by real-time B-mode imaging, and the measurement window was manually adjusted to include only homogeneous muscle tissue while avoiding visible hyperechoic intramuscular aponeuroses, connective tissue septa, and vascular structures whenever possible. ROI dimensions ranged from 12 × 12 mm to 15 × 15 mm to achieve adequate and anatomically consistent coverage of the muscle region. ROI 3 (20 × 20 mm) included all tissues from the skin surface to a depth of 2 cm, representing a composite region corresponding to the combined extent of ROI 1 and ROI 2 (Figure 2A–D). ROI 3 was positioned to exclude visible intramuscular aponeuroses and connective tissue septa within its muscular portion. When complete exclusion was not feasible because of individual anatomy, the ROI was repositioned, or its dimensions were adjusted to minimize the inclusion of these structures while preserving the predefined anatomical boundaries.

A depth of 2 cm was selected to ensure comparability with MyotonPRO measurements, as myotonometric assessment is limited to superficial tissues within approximately 2 cm from the skin surface, representing the effective range of tissues contributing to the recorded mechanical response. This enabled a more direct comparison of mechanical properties assessed by both methods [13]. For each ROI, three independent measurements were obtained, and the mean value was used for statistical analysis. Tissue stiffness was expressed as Young’s modulus (E, kPa), calculated from shear wave propagation velocity. Previous studies have demonstrated high intra-rater reliability and reproducibility of SWE measurements in lower limb muscles [10,11,14].

### 2.5. Myotonometry

Superficial muscle tissue stiffness was also assessed using a MyotonPRO device (Myoton AS, Tallinn, Estonia). The probe was positioned perpendicular to the skin over the predefined measurement site. A constant pre-load of 0.18 N was applied, followed by five brief mechanical impulses (0.4 N, 15 ms), inducing local tissue deformation. The resulting damped oscillations were recorded by an integrated accelerometer, and dynamic stiffness was calculated and expressed in N/m [15]. Measurements were repeated if the coefficient of variation (CV) exceeded 3%, following a short rest period. All assessments were performed under standardized conditions by the same experienced examiner to ensure measurement reliability.

### 2.6. Statistical Analysis

Statistical analyses were performed using PQStat software, version 1.8.6. The normality of data distribution was assessed using the Shapiro–Wilk test. Demographic characteristics and study variables were presented as mean ± standard deviation (SD) and as median with interquartile range (Q1 to Q3), as appropriate. The relationships between muscle stiffness measured using myotonometry with the MyotonPRO device (N/m) and shear wave elastography (SWE, kPa) values obtained from individual regions of interest (ROI 1, ROI 2, and ROI 3) were evaluated using Spearman’s rank correlation coefficient (ρ). Additionally, the association between muscle stiffness and subcutaneous tissue thickness (mm) was assessed. The strength of correlations was interpreted as follows: ρ < 0.20, very weak; 0.20 to 0.39, weak; 0.40 to 0.59, moderate; 0.60 to 0.79, strong; ≥0.80, very strong. To determine the independent contribution of selected variables to muscle stiffness measured using the MyotonPRO device, multiple linear regression analysis was performed. The regression model included variables potentially influencing the measurement outcome, including elastographic values from the analyzed ROIs, subcutaneous tissue thickness, and body mass index (BMI, kg/m^2^). This analysis allowed identification of independent predictors of muscle stiffness while controlling for the effects of other variables included in the model. Agreement between measurement methods was assessed using Bland–Altman analysis. Bland–Altman plots were constructed to illustrate the differences between MyotonPRO and SWE measurements in relation to their mean values. The mean difference (bias) and limits of agreement (LoA) were calculated as the mean difference ± 1.96 standard deviations (SD). This analysis enabled evaluation of agreement between the methods and identification of potential systematic measurement bias. A significance level of *p* < 0.05 was considered statistically significant.

The sample size was calculated using G*Power software (version 3.1.9.7). An a priori power analysis was performed for correlation analysis with the following parameters: test family: exact; statistical test: correlation (bivariate normal model); and type of power analysis: a priori. The analysis assumed a two-tailed test, an expected correlation coefficient of r = 0.5 based on previous studies comparing biomechanical assessment methods of skeletal muscle stiffness using myotonometry and shear wave elastography [10], a significance level of α = 0.05, and a statistical power of 80%. The required sample size was estimated at 29 participants. To increase the robustness and precision of the estimates, a larger sample was recruited. A total of 56 participants were included, all of whom completed the study protocol and were analyzed.

## 3. Results

As shown in Table 1, a total of 56 healthy young adults were included in the study. The mean age of participants was 25.9 ± 2.9 years, and the mean BMI was 24.6 ± 3.8 kg/m^2^. In elastographic analysis, the highest muscle stiffness values were observed in ROI 2, whereas lower values were recorded in ROI 3 and ROI 1, respectively. Muscle stiffness assessed using the MyotonPRO device showed moderate variability within the study population. In contrast, the thickness of superficial tissues was relatively low and exhibited limited inter-individual variability.

Table 2 summarizes the results of Spearman’s rank correlation analysis. A moderate positive association was observed between muscle stiffness measured using the MyotonPRO device and SWE values in ROI 2 and ROI 3. The strongest association was observed for ROI 3, with a comparable correlation strength for ROI 2. No significant relationship was found between MyotonPRO-derived muscle stiffness and SWE values in ROI 1, nor with superficial tissue thickness.

**Figure 3:**
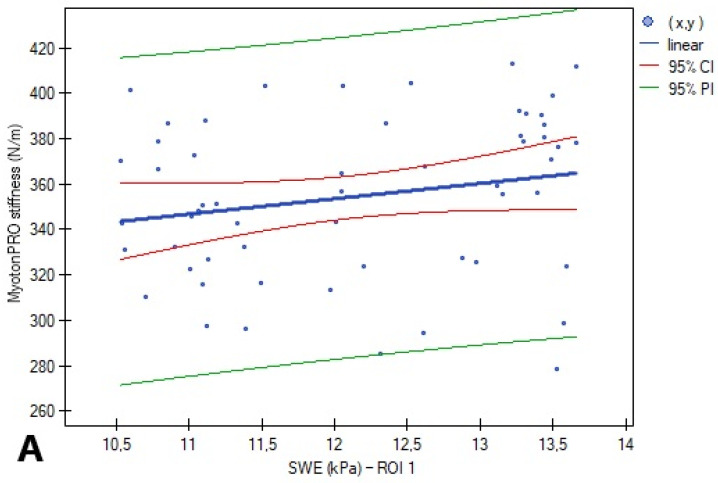

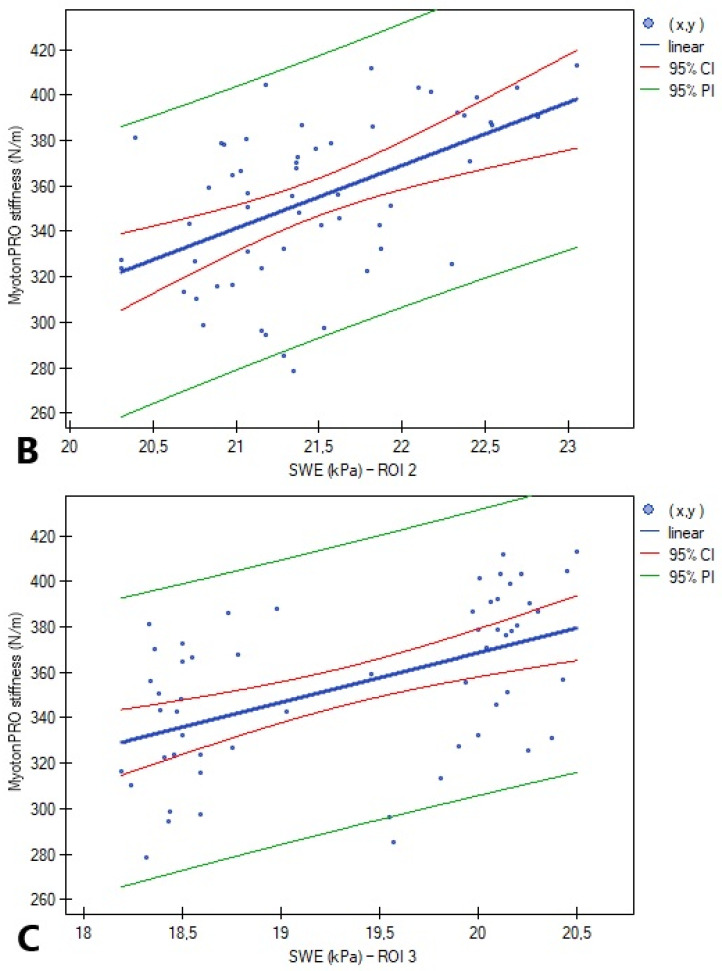
(**A**–**C**). Linear regression analysis showing the relationship between MyotonPRO-derived muscle stiffness (N/m) and shear wave elastography (SWE) measurements (kPa) in different regions of interest (ROI): (**A**) ROI 1, including superficial tissues (skin, subcutaneous tissue, and deep fascia); (**B**) ROI 2, including muscle tissue only; and (**C**) ROI 3, including all tissues from the skin surface to a depth of 2 cm. Blue dots represent individual observations (*n* = 56), the blue line indicates the fitted linear regression, the red lines represent the 95% confidence interval (CI), and the green lines represent the 95% prediction interval (PI).

As shown in Table 3 and Figure 3A–C, multiple linear regression analysis demonstrated that the overall model was statistically significant and explained approximately 38% of the variance in muscle stiffness measured using the MyotonPRO device. Among the analyzed predictors, SWE values in ROI 2 and ROI 3 were significantly associated with increased muscle stiffness. The remaining variables, including BMI, SWE values in ROI 1, and superficial tissue thickness, were not significantly associated with muscle stiffness in the model.

Visual inspection of the regression plots (Figure 3A–C) revealed differences in the distribution of data points across the analyzed regions of interest. In ROI 2, a clear positive linear relationship was observed between SWE values and muscle stiffness measured using the MyotonPRO device, with relatively low dispersion of data points around the regression line. In contrast, ROI 1 showed a weaker and non-significant trend, accompanied by greater variability of observations. In ROI 3, a greater dispersion of data points and a less homogeneous distribution relative to the regression line were observed compared with ROI 2. Despite this, the relationship between SWE and MyotonPRO-derived muscle stiffness in ROI 3 remained statistically significant. Additionally, a tendency for clustering of observations was noted in this region.

**Figure 4:**
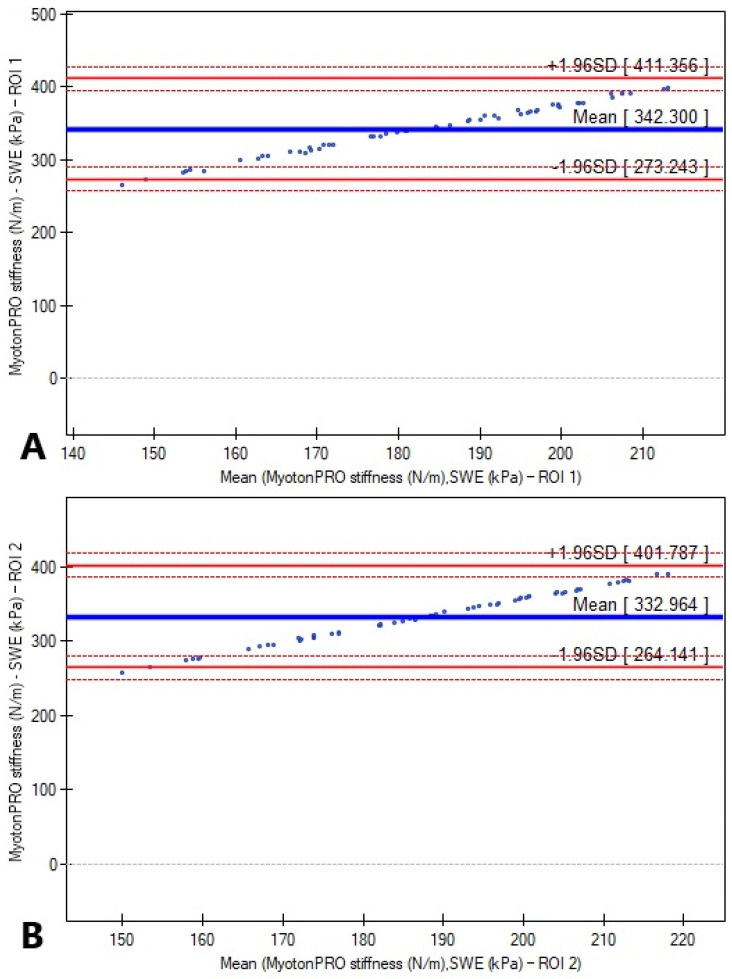

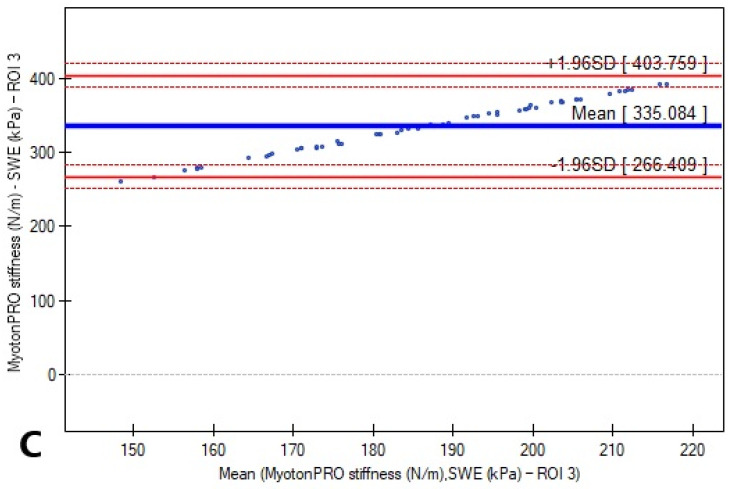
(**A**–**C**). Bland–Altman plots illustrating the agreement between MyotonPRO-derived muscle stiffness and shear wave elastography (SWE) measurements obtained from ROI 1 (**A**), ROI 2 (**B**), and ROI 3 (**C**). The *x*-axis represents the mean value of the two measurement methods, whereas the *y*-axis represents the difference between MyotonPRO and SWE measurements. The solid blue line indicates the mean bias, and the dashed red lines represent the 95% limits of agreement (mean ± 1.96 SD).

Figure 4A–C illustrates the Bland–Altman analysis demonstrating the agreement between MyotonPRO and SWE measurements. In all cases, the majority of observations fell within the limits of agreement defined as ±1.96 SD, with comparable widths of the agreement intervals across regions, approximately 137 to 138 units. The mean difference (bias) indicated a systematic shift between methods, with higher values obtained using MyotonPRO compared with SWE. Moreover, an increasing trend in differences with rising mean values was observed, suggesting the presence of proportional bias, with greater discrepancies between methods at higher levels of muscle stiffness. No substantial differences in agreement characteristics were observed between ROI 1, ROI 2, and ROI 3, as the distribution of data points and the width of the limits of agreement were similar across regions. Overall, the findings indicate moderate agreement between MyotonPRO and SWE measurements, accompanied by a systematic measurement difference and evidence of proportional bias. These results suggest that the methods are not interchangeable, but may provide complementary information regarding the mechanical properties of muscle.

## 4. Discussion

The present study demonstrates that MyotonPRO-derived muscle stiffness reflects a composite mechanical response of multiple tissue layers rather than isolated muscle properties. The moderate but consistent associations observed with SWE measurements in ROI 2 and ROI 3, together with the lack of correlation in ROI 1, indicate that deeper tissue structures, including muscle and fascia, play a dominant role in shaping the recorded mechanical response.

A key factor underlying these relationships is the tissue composition of the analyzed regions. ROI 2 and ROI 3 encompassed a depth of up to 20 mm, corresponding to the effective range of the mechanical impulse generated by MyotonPRO. Given the mean thickness of superficial tissues (4.59 mm), approximately 77% of the analyzed cross-section consisted of muscle tissue, with the remaining 23% representing superficial layers. Despite this similar depth range, ROI 2 was defined to include muscle tissue exclusively, whereas ROI 3 additionally incorporated the deep fascia. Due to its higher mechanical stiffness, even a relatively thin fascial layer may influence the overall mechanical response. This likely explains the slightly stronger association observed for ROI 3, which may better reflect the integrated biomechanical behavior captured by MyotonPRO. Differences between ROI 2 and ROI 3 were also reflected in data distribution patterns. ROI 3 demonstrated greater dispersion of data points, consistent with increased tissue heterogeneity, whereas ROI 2 showed a more homogeneous distribution. These findings suggest that correlation analysis alone may not fully capture the complexity of interactions between tissue components contributing to the measured stiffness. Notably, the regression model explained approximately 38% of the variance in MyotonPRO-derived stiffness, indicating that a substantial proportion of the mechanical response is influenced by factors not captured by SWE. This suggests that MyotonPRO may reflect additional biomechanical properties, such as viscoelastic behavior or interactions between adjacent tissue layers.

These findings have important clinical implications. Although MyotonPRO is commonly used as a surrogate measure of muscle stiffness, the present results indicate that it reflects a broader biomechanical response involving both muscle and surrounding connective tissues. This may be particularly relevant in conditions such as spasticity, myofascial disorders, or post-injury rehabilitation, where structural and mechanical changes occur across multiple tissue layers rather than within muscle fibers alone. Accordingly, changes in MyotonPRO-derived stiffness observed in these clinical conditions should not necessarily be interpreted as reflecting isolated alterations in muscle tissue. Instead, they may also represent changes in fascial and other connective tissue structures. Combining MyotonPRO with layer-specific SWE may therefore improve the interpretation of tissue-specific biomechanical alterations and facilitate more targeted rehabilitation strategies. The findings of the present study are consistent with previous research. Ren et al. [7] and Kaya Keles et al. [16] demonstrated that tibialis anterior stiffness increases with muscle elongation due to increased passive tension in muscle fibers and connective tissue structures. They also emphasized that SWE values depend on muscle architecture, fiber orientation, and local structural variability, which may influence shear wave propagation and measurement variability depending on ROI location. In the present study, measurements were performed under standardized resting conditions with the ankle positioned close to the anatomical neutral position. Participants were instructed to remain fully relaxed throughout the examination, with no voluntary muscle activation required during the measurements. Therefore, the recorded stiffness values are likely to represent the passive mechanical properties of the examined tissues rather than changes associated with muscle elongation. The importance of measurement location is further supported by the study of Le Sant et al. [17], which demonstrated significant heterogeneity in stiffness within the same muscle. Methodological factors, including probe orientation, limb positioning, and subtle changes in muscle tension, may also influence SWE results [2].

The moderate association between SWE and MyotonPRO observed in the present study is consistent with previous reports demonstrating that these methods assess related, but not identical, biomechanical properties [4,5,10,18]. The main contribution of the present study is that it extends previous observations by demonstrating, for the first time, which tissue layers underlie the relationship between MyotonPRO-derived stiffness and SWE measurements. Whereas previous studies established that these methods are correlated, our layer-specific SWE analysis indicates that MyotonPRO-derived stiffness primarily reflects the combined mechanical contribution of muscle tissue and deep fascia rather than isolated muscle tissue alone. These differences arise from their distinct measurement principles: SWE evaluates stiffness within a defined ROI based on shear wave propagation, whereas MyotonPRO reflects the response of a broader tissue complex. Consequently, systematic differences between methods may occur, as confirmed by Bland–Altman analysis, which demonstrated a consistent bias toward higher values obtained with MyotonPRO and increasing discrepancies at higher stiffness levels. The present findings are also consistent with studies showing that SWE can assess different tissue layers, although superficial measurements tend to be more reliable than deeper ones [19]. The presence of fascia and other connective structures may increase measurement heterogeneity and contribute to differences observed between ROIs.

In summary, MyotonPRO and SWE provide related but not interchangeable information on muscle mechanical properties. Regions encompassing both muscle tissue and deep fascia appear to better reflect the biomechanical response captured by MyotonPRO, highlighting the importance of ROI selection and tissue composition in the interpretation of muscle stiffness measurements.

From a diagnostic perspective, the present findings suggest that MyotonPRO measurements should not be interpreted as representing isolated muscle stiffness alone. Instead, the recorded values appear to reflect the integrated mechanical behavior of multiple tissue layers, including fascia. This observation may be particularly relevant in musculoskeletal disorders involving alterations in both muscular and connective tissues, where combined assessment using SWE and myotonometry may provide complementary biomechanical information. Overall, these findings may improve the interpretation of stiffness measurements and support more accurate application of myotonometric and elastographic methods in musculoskeletal diagnostics, rehabilitation, and research.

### Limitations of the Study

This study has several limitations that should be considered when interpreting the results. First, the study was conducted in a group of young, healthy individuals, which limits the generalizability of the findings to clinical populations and older individuals. Second, only a single model muscle was analyzed, and the results should not be generalized to muscles with different architecture and depth. Although measurement procedures were standardized, SWE results may still be influenced by technical factors such as slight variations in probe pressure, alignment with muscle fibers, and ROI placement. Additionally, the use of variable ROI sizes for superficial tissues, although anatomically justified, may have introduced minor variability in the results. Finally, MyotonPRO does not allow selective assessment of individual tissue layers, which limits the ability to determine their specific contributions to the measured mechanical response. Despite these limitations, the study provides a solid basis for further research in this area. Furthermore, the lack of a clinical population limits the direct applicability of the findings to pathological conditions. Future studies should investigate whether similar relationships are observed in patients with altered muscle and connective tissue properties. Further validation is also warranted in muscles with different anatomical depth and architectural characteristics to determine the generalizability of the present findings.

## 5. Conclusions

The present findings demonstrate that MyotonPRO-derived stiffness reflects a composite biomechanical response involving both muscle and fascial tissues rather than isolated muscle properties. The strongest associations were observed in regions encompassing muscle tissue and the muscle–fascia complex, whereas no significant relationship was identified for superficial tissues alone.

These findings emphasize the importance of tissue composition and ROI selection when interpreting stiffness measurements, indicating that MyotonPRO captures an integrated mechanical response influenced by multiple tissue layers. Consequently, MyotonPRO measurements should not be interpreted as representing isolated muscle stiffness alone.

From a diagnostic perspective, layer-specific SWE may improve understanding of the tissue structures contributing to MyotonPRO-derived stiffness and facilitate more accurate interpretation of biomechanical measurements. Combined assessment using SWE and myotonometry may provide complementary information for musculoskeletal diagnostics, rehabilitation monitoring, and future research applications.

## Figures and Tables

**Table 1:** Demographic characteristics of the study participants and mechanical properties of the examined tissues.

Variable	Mean ± SD	95% CI	Median (Q1–Q3)
Age (years)	25.93 ± 2.87	25.16–26.70	25 (24–28)
BMI (kg/m^2^)	24.63 ± 3.82	23.61–25.65	24.19 (21.60–26.36)
SWE ROI 1 (kPa)	12.14 ± 1.10	11.84–12.43	12.06 (11.09–13.29)
SWE ROI 2 (kPa)	21.47 ± 0.66	21.30–21.65	21.36 (21.02–21.86)
SWE ROI 3 (kPa)	19.35 ± 0.82	19.13–19.57	19.56 (18.50–20.12)
MyotonPRO stiffness (N/m)	354.44 ± 35.45	344.94–363.93	356.5 (326.63–382.25)
Superficial tissue thickness (mm)	4.60 ± 1.57	4.18–5.02	4.35 (3.45–5.40)

*n* = 56 for all variables. Abbreviations: BMI—body mass index; SWE—shear wave elastography; ROI—region of interest; CI—confidence interval; (Q1–Q3)—first quartile–third quartile.

**Table 2:** Spearman correlation between MyotonPRO stiffness and SWE measurements and superficial tissue thickness.

Variables	*n*	ρ	95% CI	*p*-Value
MyotonPRO stiffness—SWE ROI 1	56	0.216	−0.058–0.460	0.110
MyotonPRO stiffness—SWE ROI 2	56	0.516	0.285–0.690	<0.001
MyotonPRO stiffness—SWE ROI 3	56	0.550	0.329–0.714	<0.001
MyotonPRO stiffness—superficial tissue thickness	56	−0.212	−0.456–0.062	0.116

Abbreviations: ρ—Spearman rank correlation coefficient; CI—confidence interval.

**Table 3:** Multiple linear regression analysis for predictors of MyotonPRO stiffness.

Predictor	b	SE	95% CI	β (Standardized)	t	*p*-Value
BMI (kg/m^2^)	−0.62	1.08	−2.80–1.56	−0.07	−0.57	0.571
SWE ROI 1 (kPa)	2.67	3.73	−4.82–10.17	0.08	0.72	0.477
SWE ROI 2 (kPa)	19.88	7.03	5.75–34.01	0.37	2.83	0.006
SWE ROI 3 (kPa)	13.79	5.56	2.63–24.95	0.32	2.48	0.016
Superficial tissue thickness (mm)	−1.63	2.56	−6.76–3.51	−0.07	−0.64	0.528

Model statistics: *n* = 56, R = 0.613, R^2^ = 0.376, adjusted R^2^ = 0.313, F = 6.02, *p* < 0.001. Abbreviations: b—unstandardized regression coefficient; SE—standard error of the regression coefficient; 95% CI—95% confidence interval for the regression coefficient; β (standardized)—standardized regression coefficient; t—t-statistic for the regression coefficient.

## Data Availability

Data are available under reasonable request to the corresponding author.

## Abbreviations

SWE	shear wave elastography
ROI	regions of interest
BMI	body mass index
ICC	intraclass correlation coefficient
MSK	musculoskeletal
SD	standard deviation
